# Regenerative Management of a Late Endoperiodontal Complication After Phased Perio-Orthodontic Therapy: A Case Report

**DOI:** 10.7759/cureus.89462

**Published:** 2025-08-06

**Authors:** Satoru Morikawa, Kazuya Watanabe, Taneaki Nakagawa

**Affiliations:** 1 Department of Dentistry and Oral Surgery, Keio University School of Medicine, Tokyo, JPN

**Keywords:** enamel matrix derivative, endoperiodontal lesion, orthodontic treatment, periodontal regeneration, stage iii periodontitis

## Abstract

A man in his mid-50s with stage III periodontitis underwent a structured, multiphase treatment. Following the initial therapy that achieved periodontal stability (evidenced by improved clinical parameters), orthodontic treatment with strategic extractions was performed. Unexpectedly, a severe endoperiodontal lesion subsequently developed in the left maxillary canine. Cone-beam CT confirmed extensive circumferential bone resorption. After endodontic therapy, periodontal regenerative therapy using enamel matrix derivative and bovine bone graft was performed. Six months post-surgery, the probing pocket depth improved from 6 mm to 3 mm, with no bleeding on probing, and radiographs showed improved radiopacity. A 2.5-year follow-up demonstrated a stable periodontal condition. This case underscores that late-stage complications can arise even with thorough evidence-based therapy. This highlights the importance of proactive monitoring and demonstrates the potential for successful regeneration of severe bone defects.

## Introduction

Stage III periodontitis is characterized by extensive periodontal destruction, including significant interdental clinical attachment loss and radiographic bone loss extending to the mid-third of the root or beyond [1], and it poses a significant challenge, particularly when associated with malocclusion, often necessitating both periodontal and orthodontic interventions [2]. Orthodontic treatment is associated with an increased risk of periodontitis. Inadequate plaque control and active inflammation can exacerbate periodontal breakdown, thereby contributing to further attachment loss [2,3]. Moreover, the biomechanical principles involved in tooth movement in a reduced periodontium require cautious force application and regular monitoring [4].

Recent systematic reviews have shown the effectiveness of enamel matrix derivatives (EMD) and deproteinized bovine bone mineral (DBBM) in enhancing periodontal regeneration. A network meta-analysis [5] highlighted the long-term clinical advantages of regenerative therapies, including the combination of EMD and DBBM. Furthermore, a randomized controlled clinical trial [6] reported significant clinical improvements following the application of EMD and DBBM in non-contained intrabony defects. These results provide strong evidence that supports our approach.

## Case presentation

A man in his mid-50s, with a chief complaint of tooth mobility and gingival swelling, was referred to our periodontics department. The treatment plan consisted of comprehensive periodontal therapy to stabilize his condition, followed by adjunctive orthodontic treatment to improve occlusal function and dental alignment. The patient was a nonsmoker who consumed alcohol occasionally and had no significant medical or medication usage history.

Investigations

**Figure 1:**
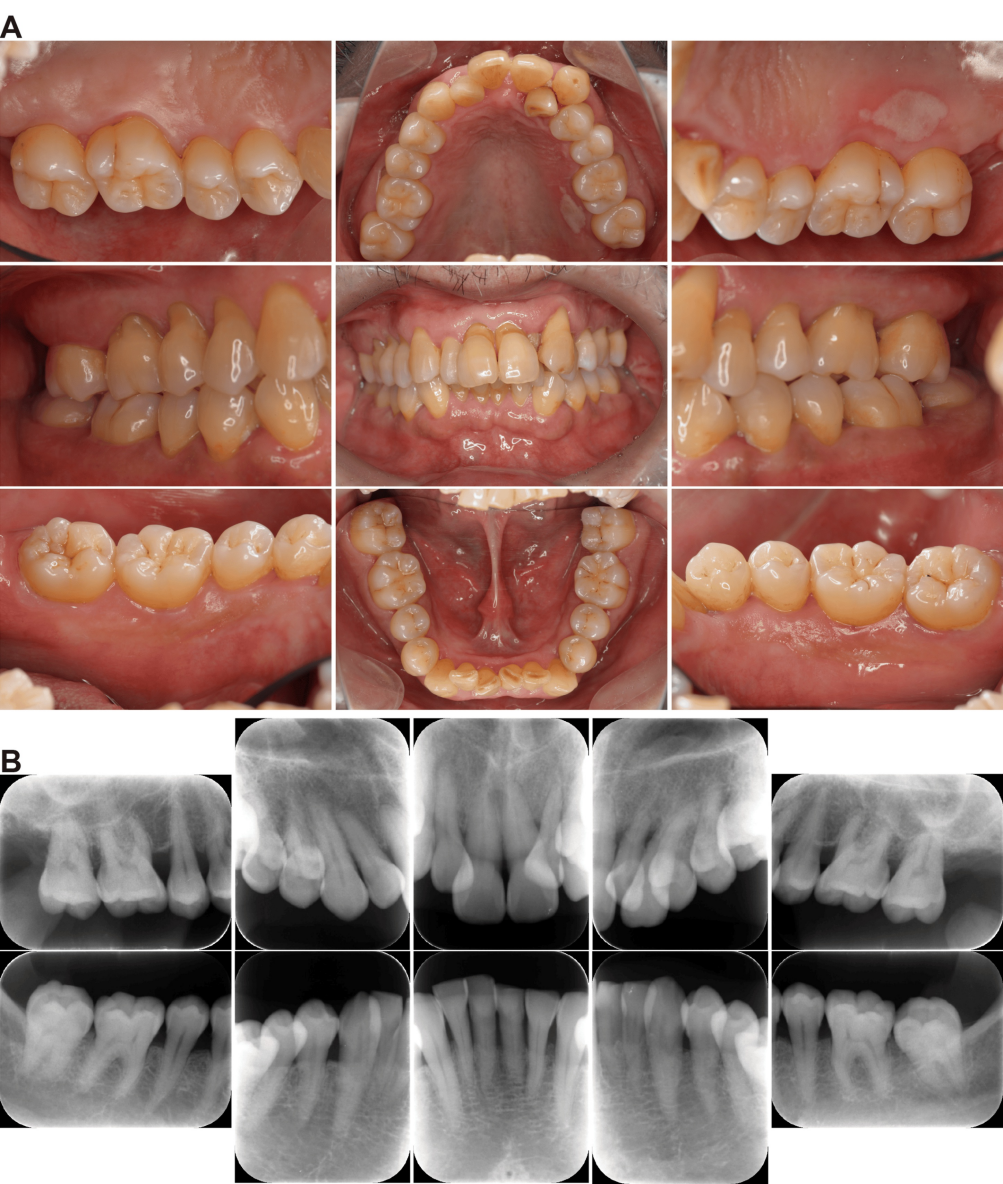
Initial patient presentation (A) Intraoral images showing generalized gingival inflammation and poor oral hygiene (PCR: 92%). (B) Dental radiographs showing generalized horizontal bone loss consistent with stage III periodontitis. PCR: plaque control record

Upon initial examination, the patient presented with poor oral hygiene, as evidenced by a plaque control record (PCR) of 92%. Clinical assessment revealed generalized gingival inflammation (Figure 1A), with a full-mouth bleeding on probing (BOP) score of 51.8%. A history of recurrent aphthous ulcers was also reported. Furthermore, several teeth exhibited non-carious cervical lesions, suggesting occlusal trauma as a contributing factor. The mean periodontal pocket depth (PPD) was 3.8 mm, with 12 sites (7.2%) exhibiting PPDs ≥6 mm. Notably, the maxillary left canine (tooth #23), which later became the focus of complications, presented with PPDs of 5 mm (mesiobuccal), 3 mm (buccal), and 2 mm (distobuccal), with BOP noted at all sites. Dental radiography revealed generalized horizontal bone loss with severe resorption in the maxillary anterior region (Figure 1B). Based on these findings, the patient was diagnosed with generalized stage III, grade C periodontitis [1].

Treatment

The treatment was performed in four distinct phases, consistent with the European Federation of Periodontology S3 level clinical practice guidelines [7,8].

Phase 1

The initial periodontal therapy, also known as the etiologic phase, aimed to control inflammation and infection. The patient was provided with comprehensive instructions on oral hygiene, followed by scaling and root planing. A formal re-evaluation was conducted three months later. The patient’s oral hygiene improved dramatically, with PCR decreasing from 92% to 19%. The BOP score also showed a significant reduction (18%).

Phase 2

Despite improvements following surgical periodontal therapy, residual deep pockets remained. Therefore, open flap debridement was performed on the maxillary right first molar (tooth #17). After the healing period, another re-evaluation confirmed further improvement in periodontal health. PCR was reduced to an excellent level (5%), indicating a high level of compliance. With the control of inflammation and stabilization of periodontal health, the patient was deemed ready for orthodontic treatment.

Phase 3

**Figure 2:**
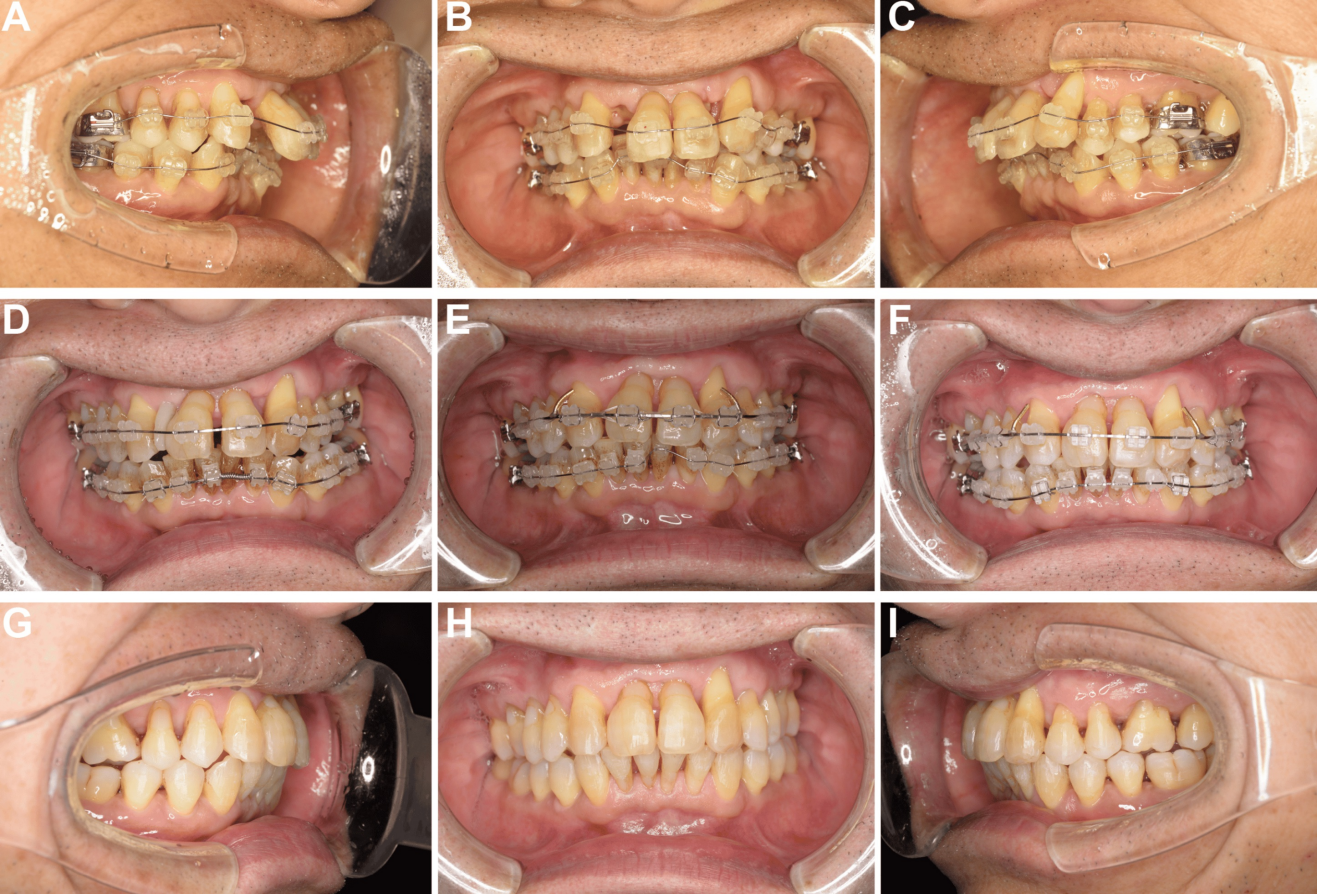
Progress in orthodontic treatment (A-C) Initial placement of orthodontic appliance. (D-F) Treatment progression showing space management. (G-I) Post-treatment images obtained after 22 months showing improved alignment and occlusion.

Orthodontic treatment began with bilateral maxillary lateral incisor extraction to address crowding. Initial leveling was performed using 0.014-inch round nickel-titanium wires (Figure 2A-2C). The wire size progressively increased (Figure 2D-2F), and routine periodontal maintenance was continued throughout the 22-month treatment period. Dental alignment and occlusion were significantly improved (Figure 2G-2I).

Phase 4

**Figure 3:**
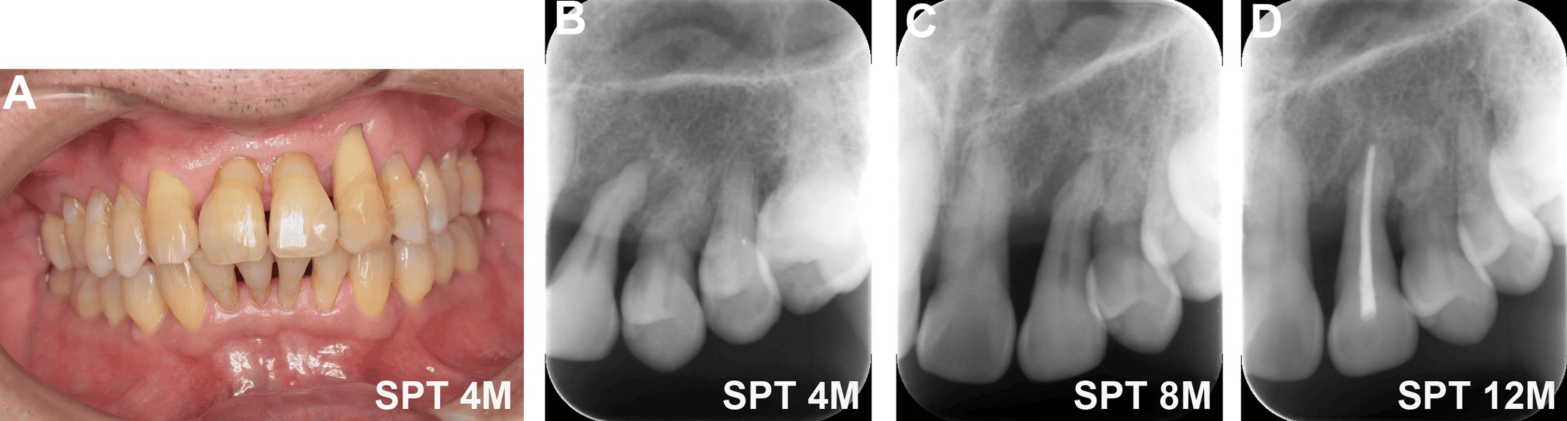
Clinical progression of periapical lesion and endodontic intervention (A) Sinus tract observed on the distal gingiva of the left maxillary canine at SPT 4M. (B-D) Periapical radiographs showing lesion development and persistence from SPT 4M to SPT 12M, despite endodontic treatment. SPT: supportive periodontal therapy

**Figure 4:**
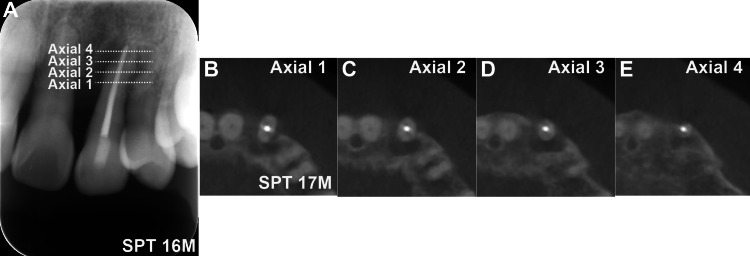
CBCT-based assessment of circumferential bone defects (A) Radiograph at SPT 16M showing persistent periapical radiolucency. (B-E) Axial CBCT images at SPT 17M revealing extensive circumferential bone loss around the maxillary canine root. CBCT: cone-beam computed tomography, SPT: supportive periodontal therapy

The patient transitioned to supportive periodontal therapy (SPT) and underwent management for complications following active orthodontic treatment. At SPT initiation (SPT 0M), the periodontal condition was stable (PCR: 11%). However, at SPT 4M, a sinus tract developed on the distal gingiva of the left maxillary canine (Figure 3A), with a 6-mm mesiobuccal pocket. Radiographs revealed periapical radiolucency (Figure 3B), which worsened with an SPT of 8M (Figure 3C). Electric pulp testing suggested pulpal necrosis, indicating a combined endodontic-periodontal lesion. Endodontic therapy was completed (Figure 3D), but a radiograph at SPT 16M showed persistent radiolucency (Figure 4A). Cone-beam CT (CBCT) at SPT 17M revealed extensive circumferential bone defects around the root (Figure 4B-4E).

Although the sinus tract persisted, there were no acute inflammatory signs, such as swelling and suppuration, and the lesion was considered clinically chronic with low activity. Therefore, although extraction was a viable option, the decision to proceed with regenerative periodontal therapy was made. This decision was based on the patient’s strong desire for tooth retention combined with the detailed results of three-dimensional CBCT, which indicated that the defect morphology was amenable to a regenerative approach.

Regenerative surgery

**Figure 5:**
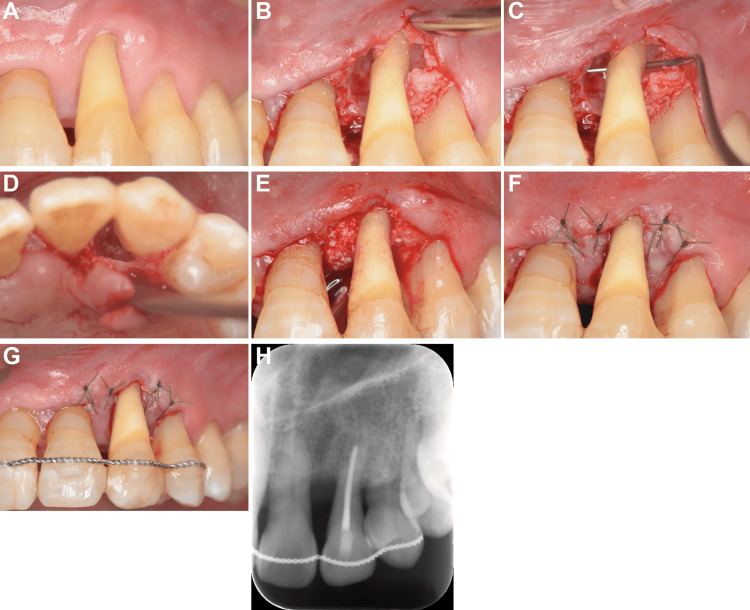
Regenerative periodontal therapy (A) Preoperative view showing the remaining sinus tract. (B-D) Intraoperative view showing the circumferential bone defect. (E) Application of EMD and DBBM. (F) Tension-free primary closure. (G) Postoperative splinting. (H) Postoperative radiograph showing radiopaque graft material filling the defect. EMD: enamel matrix derivative, DBBM: deproteinized bovine bone mineral

The sinus tract remained in the buccal gingiva of the left maxillary canine (Figure 5A). A mucoperiosteal flap was lifted, revealing a circumferential bone defect extending to the root apex (Figure 5B-5D). After debridement, periodontal regenerative therapy using a combination of EMD and DBBM was performed (Figure 5E). Written informed consent was obtained from the patient for this combination therapy, which was selected to address the severe, non-contained nature of the defect. Tension-free closure was achieved, and the treated canine was stabilized with wire splinting (Figure 5F-5G). Postoperative X-ray images showed that bone resorption around the canine teeth was adequately filled with the bone graft material (Figure 5H).

Outcome and follow-up

**Figure 6:**
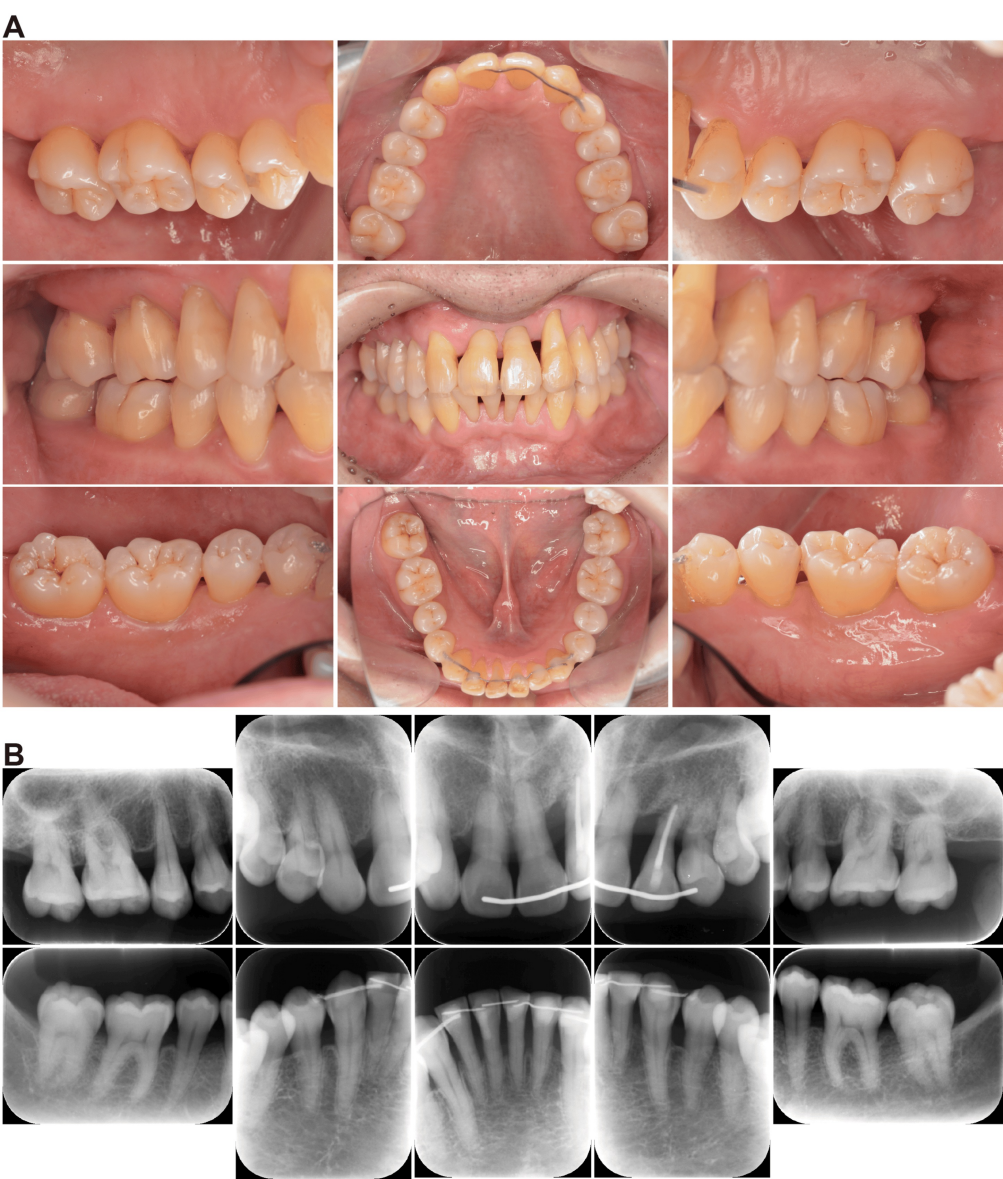
Follow-up at 2.5 years (A) Intraoral photographs confirming maintenance of periodontal health and stable dental alignment. (B) Radiographs showing stable bone levels and persistent regeneration around the treated maxillary canine.

Six months after regenerative therapy, the left maxillary canine showed significant clinical improvement, with the PPD reducing from 6 to 3 mm with no BOP, and tooth mobility eliminated (from grade 1 to 0). Radiographs revealed an increased periradicular opacity. The patient’s periodontal condition remained stable after 2.5 years (Figure 6A). He retained 26 teeth with a mean PPD of 2.1 mm, and only six sites (3.8%) showed bleeding. Radiographs showed stable bone levels around the dentition, including regenerated bone surrounding tooth #23 (Figure 6B).

## Discussion

Challenge of hidden pathogens and late-stage complications

This case report highlights a significant clinical challenge: a late-stage endo-periodontal lesion developing after an otherwise successful course of periodontal-orthodontic therapy. A literature search revealed no reports describing a similar complication under these specific circumstances, underscoring the novelty of this case. In the absence of direct precedent, we propose a pathogenic mechanism analogous to the well-documented periodontal destruction that can occur on the distal aspect of a second molar following the extraction of an impacted third molar [9,10]. We hypothesize that the etiology was multifactorial. It is plausible that despite comprehensive initial therapy, residual pathogens persisted in areas that were difficult to access. An alternative, or likely contributing, factor is occlusal trauma. The orthodontic realignment, while ultimately beneficial, may have inadvertently introduced unfavorable loading on the canine during tooth movement. This traumatic force, possibly combined with the proliferation of residual pathogens, could have predisposed the tooth to pulpal necrosis. This concept reinforces the need for proactive debridement of newly accessible areas throughout the orthodontic phase.

Successful recovery of a seemingly hopeless tooth

The second key finding was the successful preservation of a tooth with severe circumferential bone resorption extending to the apex. Such a case is often considered for extraction. The outcome in this study provides strong clinical evidence on the efficacy of combined regenerative therapies (EMD and DBBM) in treating complex, non-contained defects, a finding supported by a systematic review [5] and a randomized controlled trial [6]. CBCT was instrumental in accurately diagnosing the three-dimensional nature of the defect and confirming the potentially regenerative outcome, thereby underscoring its value in complex case management.

Clinical implications and long-term management

The primary clinical implication of this report is the need for vigilance and long-term SPT. The complication was detected early (at four months into the SPT) because of the structured follow-up schedule. For periodontally compromised patients, orthodontic treatment should be viewed as a phase in lifelong management and not as the final step [11,12]. A structured, multiphase approach that integrates periodontal, orthodontic, and regenerative therapies is essential for achieving and maintaining optimal and stable long-term outcomes. The predictability of such outcomes in middle-aged patients, like the one in this report, is a key consideration. This concern is directly addressed by a recent prospective study, which concluded that chronological aging did not significantly affect the clinical outcomes of regenerative therapy with EMD in well-maintained, non-smoking patients [13]. This suggests that the long-term stability of regenerated tissues is less dependent on age and more on adherence to a strict SPT regimen. The stable 2.5-year outcome in our case is consistent with this evidence, supporting the viability of this treatment approach in motivated older adults.

## Conclusions

A structured, multiphase approach is important for the successful management of patients with severe periodontitis receiving orthodontic treatment. Even after periodontal health stabilizes following the initial therapy, clinicians must remain vigilant, as complications such as endoperiodontal lesions can develop during long-term supportive care. This case shows that severe circumferential bone defects extending to the apex are not necessarily an indication for extraction. In particular, they can be treated effectively with combined regenerative therapies, leading to stable long-term outcomes.
